# Percutaneous A1 pulley with corticosteroid injection for trigger finger release: a systematic review

**DOI:** 10.1186/s13018-025-05776-2

**Published:** 2025-04-29

**Authors:** Jimmy Wen, Burhaan Syed, Ramy Khalil, Mouhamad Shehabat, Meraj Alam, Romteen Sedighi, Daniel Razick, Muzammil Akhtar, Adam Razick, Foad Elahi

**Affiliations:** 1 California Northstate University College of Medicine, 9700 W Taron Dr, Elk Grove, CA 95757 USA; 2 University of California, 405 Hilgard Ave, Los Angeles, CA 90095 USA; 3 California Center of Pain Medicine & Rehabilitation, 4944 Sunrise Blvd, Fair Oaks, CA 95628 USA

**Keywords:** Minimally invasive, A1 pulley, Corticosteroid, Tenosynovitis, Pain, Tendon entrapment

## Abstract

**Background:**

Corticosteroid injection (CI) is one of the first-line treatments for trigger finger (TF) before escalation to surgical procedures such as percutaneous A1 pulley (PAP) release. This systematic review compares outcomes of concurrent PAP and CI for trigger finger release (TFR).

**Methods:**

A systematic search following the Preferred Reporting Items for Systematic Reviews and Meta-Analyses (PRISMA) guidelines was conducted in PubMed, Embase, and Cochrane Library. Study variables included the number of patients, mean age, mean follow-up, affected finger, Quinnell grading, return to activity, pre-and post-operative patient-reported outcomes (PROs), and complications.

**Results:**

Seven studies were included, with 685 patients with a mean age of 52.0 years (range of 38.0 to 58.9) and a mean follow-up time of 22 weeks (range 1 week to 52 weeks). Throughout these studies, PAP and CI were performed on 243 thumbs, 115 index fingers, 189 middle fingers, 138 ring fingers, and 10 small fingers. PAP and CI reported satisfaction and pain resolution for 96.2% (five studies) of patients. Additionally, all patients returned to activity, sports, or work (three studies).

**Conclusion:**

Concurrent PAP and CI positively affect clinical outcomes, PROs, and is a well-tolerated procedure with a low rate of complications.

**Supplementary Information:**

The online version contains supplementary material available at 10.1186/s13018-025-05776-2.

## Introduction

Trigger finger (TF), or stenosing tenosynovitis, is characterized by pain and decreased function of the affected digit, most commonly in individuals in their fifth to sixth decade of life. The lifetime risk of developing a TF is approximately 2.6%, with risk factors including diabetes, carpal tunnel syndrome, rheumatoid arthritis, or other inflammatory syndromes, as well as overuse of the digit. It is predominantly thought to be caused by a size mismatch between the flexor tendon of the affected finger and the A1 pulley which is located near the palmar aspect of the metacarpophalangeal (MCP) joint [1]. Thickening and stenosis of the A1 pulley and nodules on the flexor tendon secondary to the aforementioned risk factors cause difficulty in digit movement, with subsequent pain, clicking, and popping [2].

Management of a TF commonly involves conservative measures, such as splinting, nonsteroidal anti-inflammatory drugs for pain control, or corticosteroid injections (CI). Splinting of the MCP joint has been shown to resolve symptoms in up to 65% of patients after 1 year; immobilizing the joint results in the flexor tendon causing less friction through the A1 pulley, which reduces inflammation [1]. However, splinting is less effective in patients with a more severe or long-standing disease course [1]. CI into the A1 pulley is an effective measure that reduces inflammation in the affected digit; however, this intervention is less effective in patients with long-standing disease, diabetics, or those involving multiple digits [1]. Further, recurrence of TF with steroid injection is relatively high, at up to 50% [3].

For individuals who have failed conservative management of a TF, surgery is a highly successful intervention that is often regarded as the final step in management [2, 4]. Open release of the TF is the gold standard, and involves an incision (e.g. vertical, horizontal, oblique) followed by dissection of neuro vasculature and incision of the A1 pulley to widen the space through which the flexor tendon passes; the efficacy of open release ranges from 90 to 100%, with a 3–9% recurrence rate [1–4]. Percutaneous A1 pulley (PAP) release is similar but involves the percutaneous insertion of a small instrument to cut the A1 pulley rather than an open incision. The efficacy of percutaneous release is comparable to that of open release with the benefit of being less invasive and having a decreased risk of infection; however, nerve damage is a possible complication associated with percutaneous release [4].

Previous studies have compared the efficacy of open and percutaneous release of the A1 pulley in treating TF [5]. However, fewer studies have evaluated the success rates of PAP release combined with CI. Given that CI is the most effective non-surgical intervention for TF, we aim to evaluate the efficacy of concurrent PAP release with CI for trigger finger release (TFR). We hypothesize that concurrent PAP and CI will produce high satisfaction rates, low risks of complications, and improved clinical outcomes compared to either procedure alone.

## Methods

### Search strategy

A search was performed following The Preferred Reporting Items for Systematic Reviews and Meta-Analyses (PRISMA) guidelines in PubMed, Embase, and Cochrane Library on September 15, 2024. All authors participated in identifying the articles included in the study. The following keywords were used during the search: (Percutaneous A1 Pulley) AND ((((((Steroid) OR (Injection)) OR (Trigger)) OR (Finger)) OR (Outcomes)) OR (Efficacy)).

### Article selection

In alignment with the PICOT (Population, Intervention, Comparison, Outcome, Time) framework, eligibility and search strategy were established. Patients of all ages were included. The intervention was PAP and CI for TFR within this population. If available comparative studies such as randomized controlled trials (RCTs) were included to compare PAP and CI effects with placebo and/or active comparators. The outcomes in this study were return to activity/sports/work, pre-and postoperative patient-reported outcomes (PROs), complications, and failures. Studies with any length of follow-up were included.

The inclusion criteria focused on patients who underwent a concurrent PAP and CI with reported outcomes. In contrast, the exclusion criteria consisted of patients who underwent PAP or CI alone or were a case report, cadaveric study, review, or animal study. Title/Abstract and full-text screening were conducted via a double-blinded dual-screening process in Covidence. If the decisions were not unanimous, discrepancies were resolved with a rigorous re-review. If discrepancies persisted, a third reviewer was consulted to determine the final article's inclusion or exclusion. All included studies underwent a thorough reference review to determine if there were additional studies to include. This protocol is registered into the PROSPERO database as CRD42024587109.

### Study quality and risk of bias assessment

Each article was assessed using the Methodological Index for Nonrandomized Studies (MINORS) criteria [6]. MINORS scores ranged from 0 (not reported), 1 (reported but inadequate), or 2 (reported and adequate), with a maximum score of 16 for non-comparative studies and 24 for comparative studies. Scores of 1 and 2 for seven or more Sects. (11 or more for comparative studies) were considered low risk of bias, five to six sections (nine to 10 for comparative studies) were moderate risk of bias, and four or less sections (eight or less for comparative studies) were high risk of bias. Evaluation of each article was done by two authors, individually before comparing their scores. Any discrepancies were resolved by re-reviewing the articles until a consensus was reached.

### Data extraction/analysis

Variables that were analyzed included the number of patients, mean age, mean follow-up, affected finger, Quinnell grading, return to activity, pre-and post-operative PROs, and complications. All extracted data was stored and analyzed via Google Sheets (Google Drive; Google, Mountain View, CA). Descriptive statistics (mean, percentage, standard deviations, ranges) were reported if applicable and available. Due to significant heterogeneity amongst the included studies, a meta-analysis was not performed, although originally planned.

## Results

**Fig. 1:**
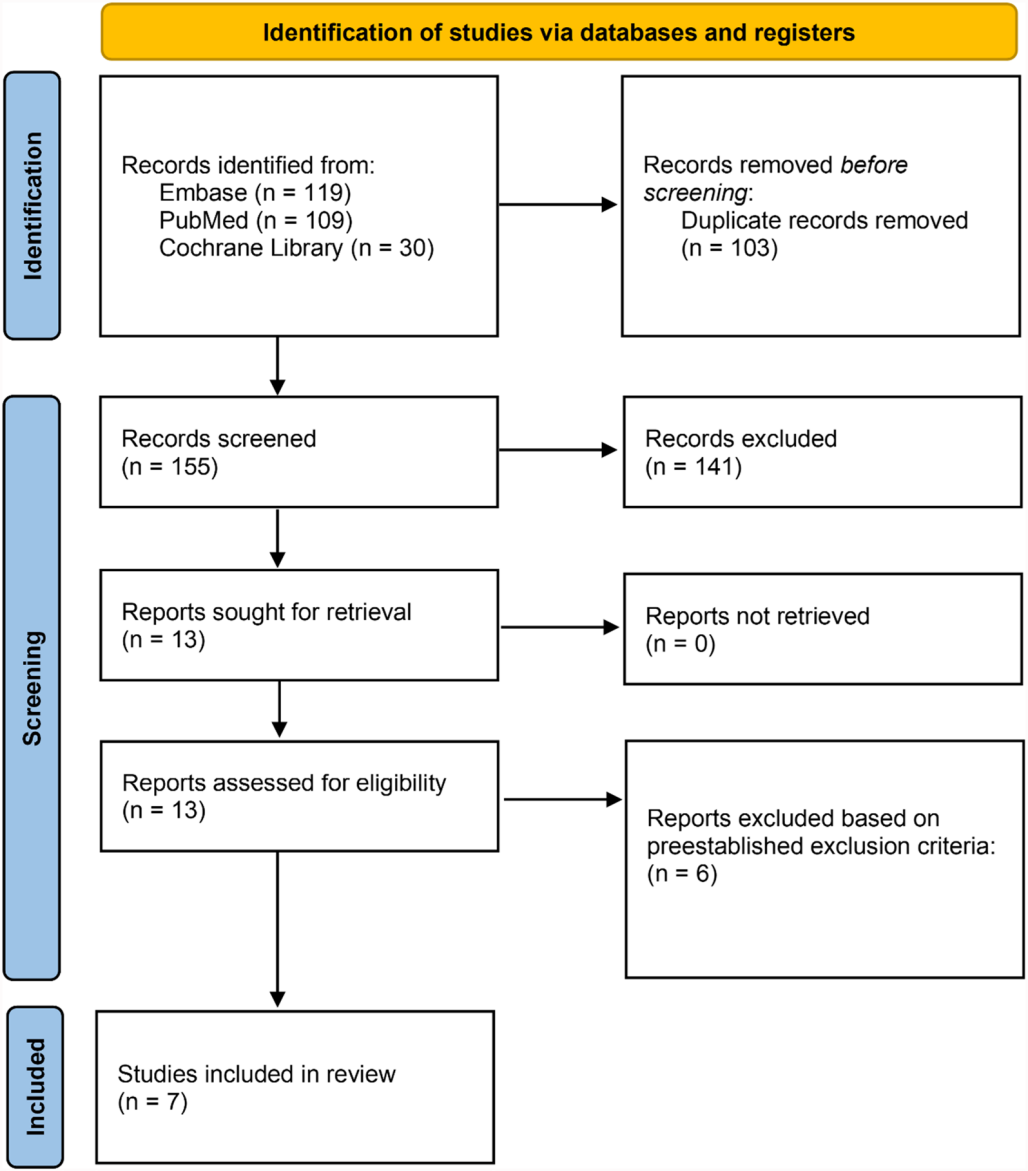
PRISMA diagram of article selection process

The initial search yielded 258 studies through Pubmed, Embase, and Cochrane Library. After the removal of 103 duplicates, 155 articles were screened by title and abstract for relevance 14 articles were screened during full-text review, and seven papers were ultimately included in this systematic review. The article selection process is further detailed in Fig. 1.

### Study characteristics

There were a total of 685 patients across all studies (32.0% male, 68% female) with a mean age of 52.0 years (range of 38.0 to 58.9) and a mean follow-up time of 22 weeks (range 1 week to 52 weeks). Of the seven studies used in this review, all seven evaluated pre and post-operative outcomes on PAP release on TFs, with and without concurrent CI [7–13]. Of the six studies that mentioned specific digits, the release was performed on 243 thumbs, 99 index fingers, 202 middle fingers, 138 ring fingers, and 10 pinky fingers [7–9, 11–13]. Zan et al. did not specify which digits comprised the 19 “other fingers” in their study [13]. The most common preoperative Quinnel grading was Grade 3, indicating triggering that can be corrected with the other hand. Corticosteroids used were triamcinolone acetonide [8, 10, 11], Betamethasone [9, 12], and a compound of betamethasone dipropionate plus betamethasone sodium phosphate [13]. The patient and study characteristics can be found in Table 1.

**Table 1:** Study characteristics and patient demographics

Author	Journal	Study year	Level of evidence (LOE)	Number of patients (M/F)	Digits	Mean age (Years)	Mean follow-up (Weeks)
Cebesoy [7]	International orthopaedics	Not reported (NR)	4	14/7	25 Thumbs	38.0	25.7
Jegal [8]	Journal of hand surgery	Jan 2013 to Jun 2014	2	20/71	Steroid Group: 19 thumb, 7 index, 15 middle, 5 ring Nonsteroid Group: 12 thumb, 8 index, 17 middle, 8 ring	58.0	12.9
Liu [9]	Kaohsiung journal of medical sciences	Jan 2013 to Dec 2013	3	Steroid group: 45/114 Nonsteroid group: 62/133	Steroid Group: 81 thumb, 25 index, 57 middle, 36 ring, 4 small Nonsteroid Group: 63 thumb, 36 index, 81 middle, 44 ring, 5 small	Steroid Group: 56.55 Nonsteroid Group: 58.91	12
Ryu [10]	Korean journal of anesthesiology	Jan 2006 to Apr 2008	3	Steroid + percutaneous release of the A1 Pulley group (Group A): 3/30 Steroid alone group (Group B): 2/34	NR	Steroid + Percutaneous release of the A1 Pulley Group: 55.6 Steroid alone Group: 54.11	52
Satish [11]	Journal of population therapeutics & clinical pharmacology	NR	3	52/28	10 thumb, 23 index, 17 middle, 30 ring	40.0	4
White [12]	Journal of medical imaging and radiation oncology	NR	4	NR	2 thumb, 2 middle, 15 ring, 1 small	NR	1
Zan [13]	Medical ultrasonography	Nov 2017 to Dec 2019	3	Steroid group: 7/18 Nonsteroid group: 8/17	Steroid Group: 16 thumb, 9 other fingers Nonsteroid Group: 15 thumb, 10 other fingers	Steroid group: 53.4 Nonsteroid group: 53.3	52

Five of seven studies were comparative [8–11, 13], with an average MINORS score of 21 (range 20 to 22) out of 24. For the other two non-comparative studies [7, 12], the mean MINORS score was 14 (range 13 to 15) out of 16. The bias risk was low in the two non-comparative studies, and moderate in the five comparative studies. MINORS scores for all studies can be found in Table S1.

### Patient-reported outcomes

Across the seven studies, parameters that were reported on were functional Visual Analogue Scale (VAS) for function, VAS for pain, modified Quinnell grade, modified patient global impression of improvement (PGI-I), and verbal numerical rating scale (VNRS). The full list of parameters and values can be found summarized in Table 2.

**Table 2:** Reported outcomes and complications/revisions of the included studies

Author	Digits	Outcomes	Post-operative value	*P* value	Complications	Revisions/ reoperation
Cebesoy [7]	25 thumbs	Mean functional VAS	2.19	< 0.001*	None	None
Jegal [8]	Steroid Group: 19 thumb, 7 index, 15 middle, 5 ring Nonsteroid Group: 12 thumb, 8 index, 17 middle, 8 ring	Mean pain VAS	Nonsteroid group: 1 Steroid Group: 2	< 0.05* for both groups 0.04**	None	NR
		Mean modified Quinnell Grade	Nonsteroid group: 1 Steroid Group: 2	N.S**		
		Mean Modified PGI-I	Nonsteroid group: 4 Steroid Group: 4	N.S**		
Liu [9]	Steroid Group: 81 thumb, 25 index, 57 middle, 36 ring, 4 small Nonsteroid Group: 63 thumb, 36 index, 81 middle, 44 ring, 5 small	NR			Steroid Group: Surgical site infection (1)	Steroid Group: Finger reoperations (4) Non Steroid Group Finger reoperations (2)
Ryu [10]	NR	VNRS	Steroid + Percutaneous release of the A1 Pulley Group (Group A): 0.39 Steroid alone Group (Group B): 1.03	< 0.05* (both Group a and B) < 0.05**	None	None
Satish [11]	10 thumb, 23 index, 17 middle, 30 ring	NR			With Steroids: Pain (5), Erythema (9), Stiffness (4) Without Steroids: Pain (9), Erythema (15), Stiffness (6)	None
White [21]	2 thumb, 2 middle, 15 ring, 1 small	NR			With steroid: Radial digital nerve neuropraxia (1) Without steroid: non-specific pain 1-week post op (2)	NR
Zan [13]	Steroid Group: 16 thumb, 9 other fingers Nonsteroid Group: 15 thumb, 10 other fingers	VAS	NR		With steroid: Finger swelling (2), finger numbness (1) Without Steroid: Finger swelling (4), finger numbness (1)	None
		PGI-I	NR			

*From baseline **Between groups

NR, not reported; VNRS, verbal numerical rating scale; VAS, Visual analog scale; PGI-I, patient global impression of improvement

In Cebesoy et al., VAS for function and relief of symptoms were reported for 21 patients. VAS for function before the operation was 26.2 and significantly decreased to 2.2 (*p* < 0.001) after 6 months. All patients reported satisfaction with the results of the surgery at the end of six months as well [7]. Liu et al. showed similar patient satisfaction with pain, reporting 97.5% of patients in the steroid group and 99.1% of those in the nonsteroid group to be pain-free and full range of motion, with no significant difference between the two groups. They also reported a significantly lower extensor lag rate after one week in fingers in the steroid group (5.4%) versus fingers in the nonsteroid group (12.7%). They further stratified extensor lag rates for each finger, but no significant findings were found between individual fingers between the groups [9]. Another study reported that 93.5% in the steroid group and 57.6% in the nonsteroid group had a complete resolution of triggering [10]. Satish et al. showed a 90% very satisfactory result at the end of four weeks with the steroid group and 72.5% with the nonsteroid group [11]. White et al. showed 100% satisfaction for both groups [12].

Only three of seven studies reported on return to work, sports and/or activity [7, 9, 11]. Of the studies that reported on return to activity, a 100% (181 patients) return rate was seen, with both steroid and nonsteroid groups [7, 11]. Satish et al. report that all patients returned to activity within a week [11]. Cebosy et al. reported all 21 patients returned to normal sports and work at the follow-up [7]. Finally, Liu et al. reported that the steroid group returned to normal work after a mean of 1.6 days, while the nonsteroid group returned to work after 1.7 days [9].

### Complications

All of the studies reported complications (685 patients), with 60 (8.8%) complications being noted [7–13]. Out of the 60 complications, 23 (38.3%) were reported in the group with concurrent steroid injections, while 37 (61.7%) were reported in non-steroid groups. In the steroid group, there was 1 surgical site infection (0.1%), 9 erythema (15%), 5 pain (8.3%), 4 stiffness (6.7%), 1 radial digital nerve neuropraxia (0.1%), 2 finger swelling (3.3%), and 1 finger numbness (1.7%). In the non-steroid group, there were 15 erythema (25%), 11 pain (18.3%), 6 stiffness (10%), 4 finger swelling (6.7%), and 1 finger numbness (1.7%). A majority of these complications were resolved after one week. No major complications were reported. For revisions/reoperations, out of five studies (574 patients), there were 6 revisions (0.1%) due to persistent range of motion limitations, with 4 (66.7%) in the steroid group and 2 (33.3%) in the non-steroid group [7, 9–11, 13]. The full details can be found in Table 2.

## Discussion

This systematic review analyzed seven studies, evaluating the effects of concurrent PAP and CI for TFR. Through the analysis of pre and post-procedural outcomes and rates of complications, PAP and CI have a positive impact on TF symptoms, and clinical outcomes, and are associated with low rates of complications.

CI is generally accepted as one of the first-line treatments for TF, with a success rate in the literature reported to range from 60 to 90% [14]. However, treatment protocols for CI vary widely with the choice of corticosteroid, technique, image guidance, and criteria for repeating the injection versus opting for surgical release. Corticosteroid choice is important in terms of their solubility, potency, and half-life. For example, triamcinolone and methylprednisolone have lower potency and half-life than betamethasone [14]. Triamcinolone has been associated with requiring additional injections compared to others in its class [15]. Additionally, it tends to concentrate around the tendon and form crystals which can affect the range of motion [13]. Contrarily, water-soluble betamethasone rarely forms crystals [13]. Methylprednisolone shows a dose-dependent improvement in outcomes and has been associated with increased need for surgical release and failure rates [14]. The efficacy of CI also increases with subsequent injections which can help offset the need for surgical intervention, with a median time from failure after CI ranging from 371 to 407 days [14]. There is also a cost-effectiveness benefit of attempting a maximum of three CI in the case of inadequate response before surgical release [16]. Outcomes of CI also vary depending on the digits, with the thumb having better outcomes for initial and repeat injections. Dala-Ali et al. found that among 90 digits with a minimum one-year follow-up a success rate of 92% in the thumb compared to 66% across the other digits (*p* = 0.001) [17].

In a 2022 meta-analysis of RCTs, at a 12-month follow-up, CI was significantly more effective in treating TF symptoms and had a lower failure rate (36.3%) than non-steroidal anti-inflammatory drugs (70.6%), and the control lidocaine injections groups (72.5%) with a *p* value of 0.0028 [14]. The risk reduction ratio also showed that CI reduced the failure rate by 50.9% compared to the control group. The pooled relative risk (RR) for treatment success was also higher for the CI group (2.64) compared to the control group (*p* < 0.001). There was also a significantly decreased need to progress to surgical release for those treated with CI compared to lidocaine injections [14]. These findings are supported by a 2018 meta-analysis showing a superior efficacy of CI compared to other non-surgical treatments (RR success rate: 1.54, 95% CI 1.02 to 2.35) but inferior to surgery (RR success rate: 0.55, 95% CI 0.48 to 0.63) [18]. However, the relapse rate was the highest with CI compared to all other treatments (RR: 19.53, 95% CI 6.23 to 61.19) [18]. A long-term study with 71 digits and a median follow-up of 8 years (7 to 8.3) found complete remission of TF symptoms in 69% of the cases without complications. Similarly, the thumb had a high success rate at 81% compared to 56% in the other digits [19].

If CI treatment fails, surgical intervention is warranted and excellent outcomes have been demonstrated with open and percutaneous approaches [5]. Open treatment is associated with increased rates of complications, slower recovery of range of motion, and scarring due to a larger incision [20]. On the other hand, percutaneous treatment is associated with iatrogenic nerve injury, incomplete release, and failed treatment leading to conversion to open release [20]. However, the percutaneous approach has several benefits, notably a shorter operation time, quicker return to activity, and lower cost [13]. A 2024 meta-analysis found no significant difference between open and percutaneous release rates regarding revision procedures, complications, or postoperative pain [5]. A 2014 meta-analysis found across 2,114 PAP procedures, the total success rate was 94% with a statistically significant trend toward improved overall success rates over time [21]. Similarly, a 2023 systematic review supported these findings with an overall success rate of 97% (n = 749) [22]. Wang et al. also found fewer failures (relative risk: 0.07, 95% CI 0.02 to 0.21) and greater levels of satisfaction (relative risk: 2.01, 95% CI 1.62 to 2.48) for PAP compared to CI [23]. Thus, percutaneous release can be a viable option to reduce the complications associated with larger skin incisions with open release.

Despite the satisfactory results with a percutaneous release, patients can present with post-operative swelling, pain, and stiffness which can potentially be ameliorated with concurrent CI [8]. In the study by Jegal et al., patients in the PAP with CI group had a greater subjective feeling of symptom improvement than the PAP-only group even though both groups had similar pain scores. Liu et al. noticed that their cohort of PAP and CI patients had a subset of patients who had a restricted range of motion but had gradual restoration over time. These findings were postulated from incomplete release, chronic flexor tendon adhesion, or post-procedure inflammation [9]. Following TFR, over-reactive local inflammation can lead to tendon adhesion and scar formation [8]. Post-procedure CI can reduce the local inflammation and pain levels and possibly soften the pulley [13].

Nakagawa et al. found with PAP, a total of 23 minor complications across 749 procedures, consisting of 4 hematomas, 15 persistent pain, and 4 transient numbness, without any major complications reported [22]. Several complications reported with CI are osteomyelitis, cellulitis, tendon rupture, fat tissue and local skin atrophy, and hypopigmentation [14]. Wang et al. found no difference in rates of complications between PAP and CI (relative risk: 3.19, 95% CI 0.51 to 19.91) [23]. Iatrogenic nerve injury in the literature has been reported to range from 1 to 5.7% [12, 24]. The thumb is more susceptible to injury because the neurovascular bundle runs closer to the pulley than the other digits [12]. Additionally, the angle of the thumb at 90 degrees to the palm makes the procedure more technically difficult. To decrease the rates of complications, it is key to inject into the tendon sheath accurately which can include image-guided modalities for accurate localization. However, other factors such as repeat injections, dosage, and comorbidities (diabetes, palpable nodule, multiple trigger fingers) can affect the complication rate [14, 18].

PAP with concurrent CI is an effective method for TFR with high success rates and low rates of complications. It is also important to note that the success rate without complications of PAP with or without CI depends on the individual’s technique and experience. This highlights the importance of attention to detail for the procedure and utilizing image-guided release if needed [10]. Other therapeutic alternatives to CI have also been trialed such as using platelet-rich plasma (PRP) for TF. At a 3-month follow-up, a 63-year-old woman experienced complete resolution of triggering with no symptom recurrence and may be explored further with larger studies [25]. PRP is effective in treating other tendon pathologies and its benefit over CI is as a potential longer-term solution [26]. Future studies should explore the optimal choice of corticosteroids for TFR and other longer-term alternatives to CI. Additionally, future studies should investigate the association between Quinnell grades, A1 pulley thickness, and TF symptoms. High-quality studies with a control or comparator group can also better control for confounding factors (eg. working conditions, activity levels, rest, climate, environment, psychological mood) that may affect patient recovery. Analyzing the outcomes and complications for each digit as well will be important in guiding clinical decision-making.

These results must also be interpreted within the context of its limitations. First, there was a wide range of follow-ups, which can affect success rates and compilation rates reported. Second, there was baseline patient demographic heterogeneity, potentially biasing the results seen across the studies. Third, the corticosteroid choice, dosage, and properties of each corticosteroid can also affect the results. Thus, the results observed must be interpreted within the context of each corticosteroid used. Fourth, the majority of included studies did not include a comparator group, thus preventing the definitive conclusion of the effectiveness of concurrent PAP and CI compared to each procedure alone. Fifth, the studies did not report outcomes based on the digit the procedure was performed on, preventing the comparison of effects by digit.

## Conclusion

Concurrent PAP and CI positively affect clinical outcomes, PROs, and well-tolerated procedures with a low rate of complications. Future studies should focus on conducting high-quality studies with control and/or active comparator groups to better determine the effectiveness of this combined procedure compared to each procedure alone.

## Supplementary Information

Below is the link to the electronic supplementary material.

Supplementary file1 (DOCX 16 KB)

## Data Availability

The datasets used and/or analyzed in the current study are available upon reasonable request. Please contact J.W. to request data from the study.

## Abbreviations

TF: Trigger finger
MCP: Metacarpophalangeal
CI: Corticosteroid injection
PAP: Percutaneous a1 pulley
TFR: Trigger finger release
PRISMA: Preferred reporting items for systematic reviews and meta-analyses
PICOT: Population intervention, comparison, outcome, time
RCTs: Randomized controlled trials
PROs: Patient-reported outcomes
MINORS: Methodological index for nonrandomized studies
VAS: Visual analogue scale
PGI-I: Patient global impression of improvement
VNRS: Verbal numerical rating scale
RR: Relative risk
