# Supplementary material for: Percutaneous A1 pulley with corticosteroid injection for trigger finger release: a systematic review
Source: J Orthop Surg Res. 2025 Apr 29;20:431. doi: 10.1186/s13018-025-05776-2 (PMC12042385; doi:10.1186/s13018-025-05776-2)
Supplement: Supplementary file 1 — Supplementary file1 (DOCX 16 KB) [file 13018_2025_5776_MOESM1_ESM.docx]

| **Author** | **Clearly stated aim** | **Inclusion of consecutive patients** | **Prospective collection of data** | **Endpoints appropriate to the aim of the study** | **Unbiased assessment of the study endpoint** | **Follow-up period appropriate to the aim of the study** | **Loss to follow up less than 5%** | **Prospective calculation of the study size** | **An adequate control group** | **Contemporary groups** | **Baseline equivalence of groups** | **Adequate statistical analyses** | **Total** |
| --- | --- | --- | --- | --- | --- | --- | --- | --- | --- | --- | --- | --- | --- |
| Cebesoy 2007 | **2** | **2** | **2** | **2** | **2** | **2** | **2** | **1** | **-** | **-** | **-** | **-** | **15** |
| Jegal 2019 | **2** | **2** | **2** | **2** | **2** | **2** | **2** | **2** | **0** | **2** | **2** | **2** | **22** |
| Liu 2016 | **2** | **2** | **2** | **1** | **1** | **2** | **2** | **2** | **0** | **2** | **2** | **2** | **20** |
| Ryu 2009 | **2** | **2** | **2** | **2** | **2** | **2** | **2** | **2** | **0** | **2** | **2** | **2** | **22** |
| Satish 2024 | **2** | **2** | **2** | **1** | **1** | **2** | **2** | **2** | **0** | **2** | **2** | **2** | **20** |
| White 2021 | **2** | **1** | **2** | **1** | **1** | **2** | **2** | **2** | **-** | **-** | **-** | **-** | **13** |
| Zan 2023 | **2** | **2** | **2** | **2** | **2** | **2** | **2** | **2** | **0** | **2** | **2** | **1** | **21** |

Table S1: Methodological quality and risk of bias
